# Redesign of a novel d-allulose 3-epimerase from *Staphylococcus aureus* for thermostability and efficient biocatalytic production of d-allulose

**DOI:** 10.1186/s12934-019-1107-z

**Published:** 2019-03-25

**Authors:** Zhangliang Zhu, Dengke Gao, Chao Li, Ying Chen, Menglu Zhu, Xin Liu, Masaru Tanokura, Hui-Min Qin, Fuping Lu

**Affiliations:** 1 Key Laboratory of Industrial Fermentation Microbiology of the Ministry of Education, Tianjin Key Laboratory of Industrial Microbiology, College of Biotechnology, National Engineering Laboratory for Industrial Enzymes, Tianjin University of Science and Technology, Tianjin, 300457 People’s Republic of China; 2 Laboratory of Basic Science on Healthy Longevity, Department of Applied Biological Chemistry, Graduate School of Agricultural and Life Sciences, The University of Tokyo, 1-1-1 Yayoi, Bunkyo, Tokyo, 113-8657 Japan

**Keywords:** d-Allulose 3-epimerase, Structural analysis, TIM-barrel fold, Site-directed saturation mutagenesis, Thermostability

## Abstract

**Background:**

A novel d-allulose 3-epimerase from *Staphylococcus aureus* (SaDAE) has been screened as a d-allulose 3-epimerase family enzyme based on its high specificity for d-allulose. It usually converts both d-fructose and d-tagatose to respectively d-allulose and d-sorbose. We targeted potential biocatalysts for the large-scale industrial production of rare sugars.

**Results:**

SaDAE showed a high activity on d-allulose with an affinity of 41.5 mM and catalytic efficiency of 1.1 s^−1^ mM^−1^. Four residues, Glu146, Asp179, Gln205, and Glu240, constitute the catalytic tetrad of SaDAE. Glu146 and Glu240 formed unique interactions with substrates based on the structural model analysis. The redesigned SaDAE_V105A showed an improvement of relative activity toward d-fructose of 68%. The conversion rate of SaDAE_V105A reached 38.9% after 6 h. The triple mutant S191D/M193E/S213C showed higher thermostability than the wild-type enzyme, exhibiting a 50% loss of activity after incubation for 60 min at 74.2 °C compared with 67 °C for the wild type.

**Conclusions:**

We redesigned SaDAE for thermostability and biocatalytic production of d-allulose. The research will aid the development of industrial biocatalysts for d-allulose.

**Electronic supplementary material:**

The online version of this article (10.1186/s12934-019-1107-z) contains supplementary material, which is available to authorized users.

## Background

Rare sugars are very useful additives and compounds in foods and pharmaceuticals. The chemical synthesis of rare sugars yields undesired by-products, and generates excessive pollution due to the many reactions and functional group protection–deprotection steps that are needed [1]. By contrast the biosynthesis of rare sugars by enzymatic pathways using Izumoring strategy is more environmental friendly, with moderate reaction conditions and high specificity, which makes them sustainable. Ketose 3-epimerases are irreplaceable in the bio-transformation of rare sugars. Enzymes from the d-tagatose 3-epimerase (DTE) and d-allulose 3-epimerase (DAE, also named as DPE) families can catalyze the reversible conversion of d-tagatose and d-fructose into respectively d-sorbose and d-allulose. Moreover, l-ribulose 3-epimerases (LRE) were found to be able to convert d-fructose for d-allulose production [2, 3]. Some genes encoding DTE/DAE/LRE family enzymes have been identified in *Agrobacterium tumefaciens* [4], *Arthrobacter globiformis* [5], *Clostridium cellulolyticum* [6], *Clostridium scindens* [7], *Desmospora* sp. [8], *Flavonifractor plautii* [9], *Pseudomonas cichorii* [10], *Rhodobacter sphaeroides* [11], *Sinorhizobium* sp. [12] and et al. However, only the crystal structures of *C. cellulolyticum* DAE [13], *A. tumefaciens* DAE [14], *A. globiformis* LRE [15], and *P. cichorii* DTE [16] have been solved.

DTE/DAE/LRE family enzymes depend on Mn^2+^ or Co^2+^ ions as cofactors [4, 6]. A C3–O3 proton-exchange mechanism was proposed in which a *cis*-enediolate intermediate is generated via O-3 when one Glu removes a proton from C-3, after which another Glu protonates C-3 from the opposite side [14, 16]. The metal-binding sites and catalytic residues of DTE, DAE, and LRE, are completely conserved implying that they belong to the same superfamily, in spite of their different substrate specificities.

On the other hand, protein engineering is a robust strategy for the creation of enzyme variants for novel biotechnological applications [17, 18]. Rational design and function modification based on structure–function relationships has enabled a significant improvement of enzymatic performance in many cases [19, 20]. Bosshart et al. reported that interactions between the subunits in multimeric proteins can be very important to enhance the thermostability and structural stability of the whole protein [21]. Zhang et al. improved both the catalytic activity and thermostability of the *C. bolteae* DAE by site-directed mutagenesis around the substrate-binding pocket [22]. These strategies have the potential to introduce the biocatalytic production of rare sugars into the realm of commercial-scale manufacture. Therefore, we selected the point mutation around its tetrameric interface and the substrate-binding pocket.

A novel d-allulose 3-epimerase from *Staphylococcus aureus* (SaDAE) has been identified as belonging to the d-allulose 3-epimerase family enzyme based on its high specificity for d-allulose. Structural modeling and rational design of functionally improved SaDAE is necessary to fully understand the DAE/DTE enzyme family. Therefore, we redesigned SaDAE for thermostability and biocatalytic production of d-allulose. The research will aid the development of industrial biocatalysts for d-allulose.

## Results and discussion

### Multiple sequence alignment

The conserved amino acids were displayed by the multiple sequence alignment among d-allulose 3-epimerases, l-ribulose 3-epimerases, and d-tagatose 3-epimerases from various strains characterized previously. SaDAE showed 18–29% with DAEases, 25–30% with DTEases. Surprisingly, SaDAE exhibited a higher sequence identity (42%) with LREase from *Mesorhizobium loti*, although it was defined as DAEases. The residues, which are responsible for metal coordination, are conserved (Additional file 1: Figure S1).

### Characterization of SaDAE

Recombinant SaDAE was successfully overexpressed in *E. coli* BL21(DE3). After cell lysis, the supernatants were purified using Ni–NTA Superflow resin followed by anion-exchange chromatography (Additional file 1: Figure S2a). Size-exclusion chromatography of purified SaDAE, with a Superdex 200 Increase 10/300 GL column, resulted in a peak between protein markers conalbumin (75.0 kDa) and aldolase (158.0 kDa), implying that SaDAE is a tetramer (Additional file 1: Figure S2b). The molecular mass was further analyzed by MALDI-TOF, revealing a protein of 134.13 kDa (Additional file 1: Figure S2c), which was four-fold higher than the predicted molecular mass of recombinant SaDAE (33.37 kDa), together with 21 residues (MGSSHHHHHHHSSGLVPRGSH) at the N-terminus. Therefore, the recombinant enzyme is a tetramer, in agreement with what was observed for the enzymes for *C. cellulolyticum* H10 [6], *A. tumefaciens* [4], and *Clostridium* sp. [23].

The secondary structure of SaDAE was evaluated using CD spectroscopy (Additional file 1: Figure S3), with a positive absorption peak at 195 nm. The percentages of α-helix, β-sheet structures, and unstructured regions were 34.8%, 10.4%, and 24.6%, respectively. The reaction products were determined by HPLC, which revealed single peaks of four products, d-allulose, d-tagatose, d-fructose, and d-sorbose at 12.48, 17.0, 18.32, and 20.37 min, respectively (Additional file 1: Figure S4).

**Fig. 1:**
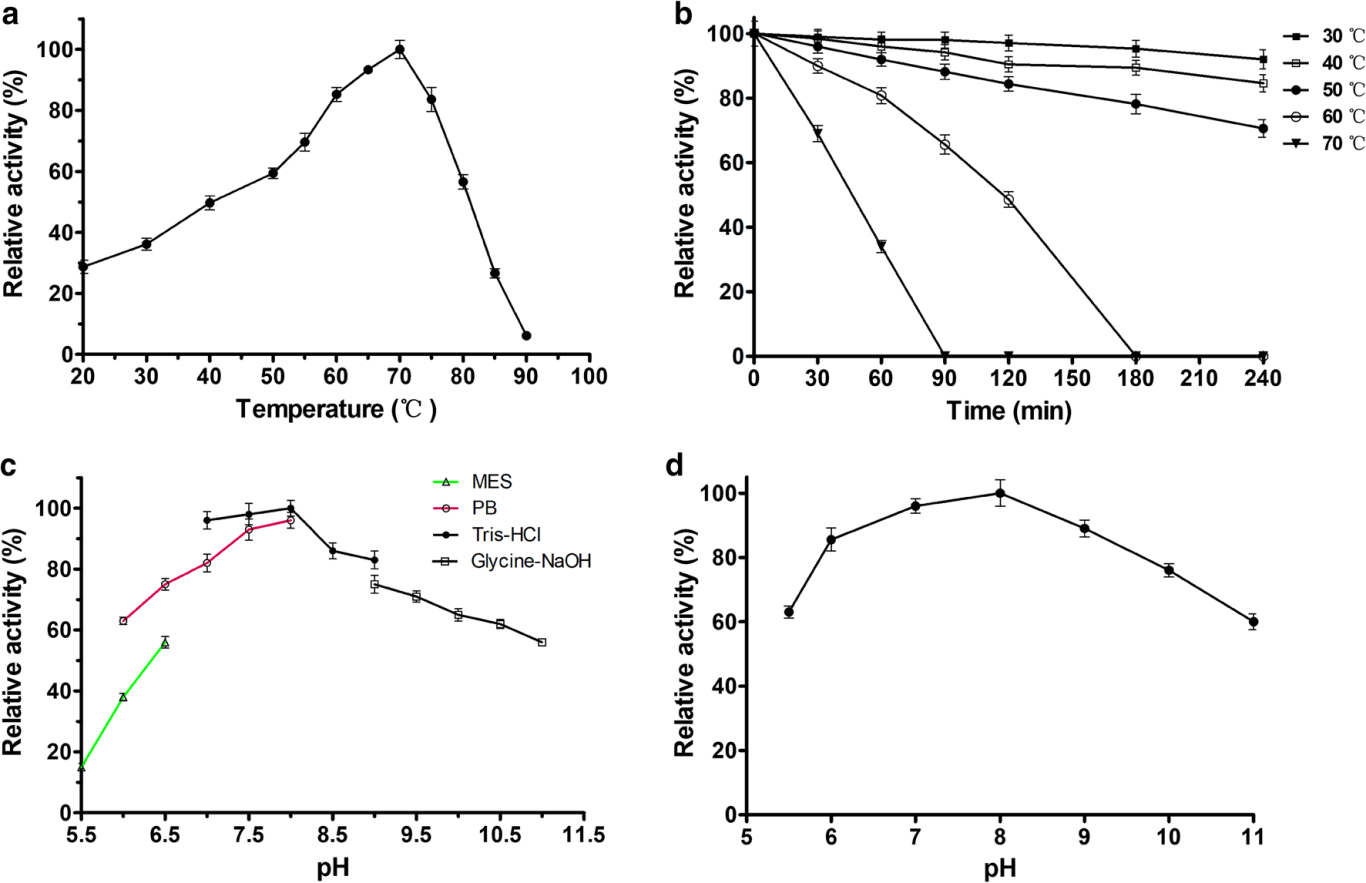
Effect of temperature and pH on activity of SaDAE. **a** temperature dependence. **b** thermostability analysis. **c** pH dependence. **d** pH stability. The activity of purified SaDAE using ÄKTA system was determined in standard assay conditions as control. All assays were repeated three times, and the data are shown as mean ± S.D

**Fig. 2:**
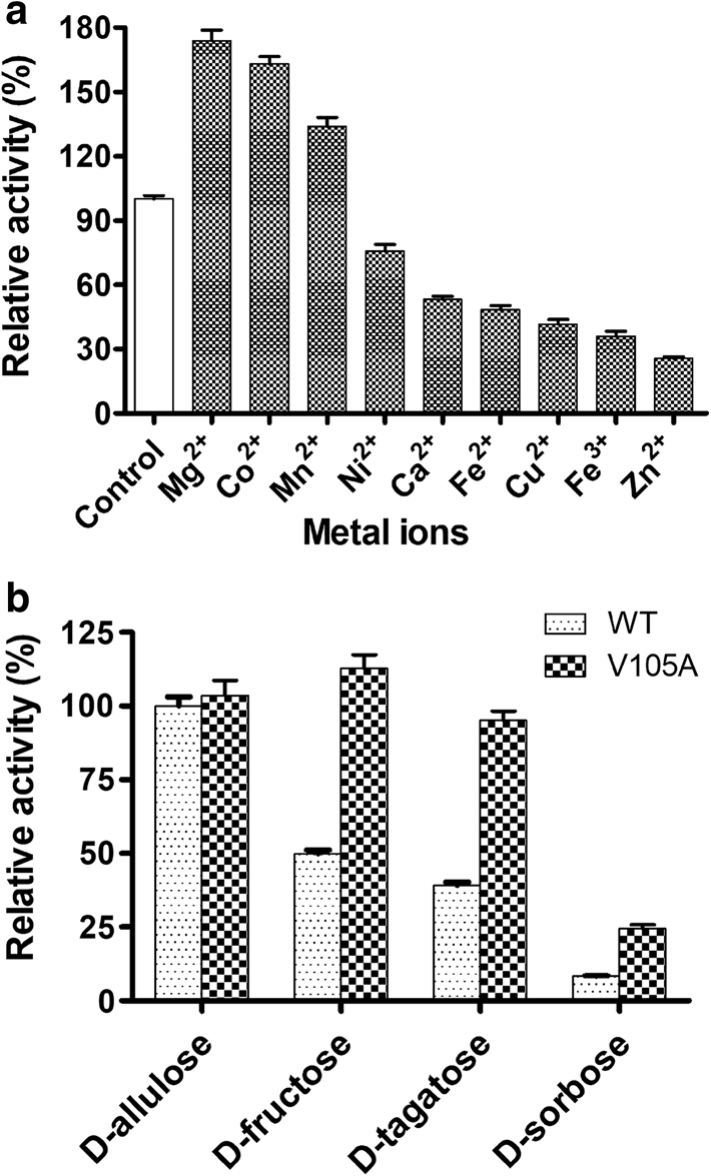
The effect of mental ions on activity of SaDAE (**a**) and substrate specificity of SaDAE WT and V105A (**b**)

The catalytic activity of SaDAE was dependent on pH and temperature conditions. The temperature-activity profile for SaDAE shown in Fig. 1a revealed an optimum temperature of 70 °C. The enzyme showed over 80% catalytic activity between 60 and 75 °C, but the activity significantly decreased above 75 °C (Fig. 1a). The enzyme was stable from 30 to 50 °C for 240 min, but the relative activity decreased to 65.6% after the enzyme was incubated for 90 min at 60 °C. Furthermore, the enzyme was inactive at 70 °C for 90 min (Fig. 1b). Compared with the family enzymes, The DAEases from *A. tumefaciens* [4], *C. cellulolyticum* [6], and *C. scindens* [7] were relatively stable below 45/50 °C. However, the relative activity declined dramatically from 55 °C at the incubation time of 30 min. Furthermore, DAEase from *Desmospora* sp. was inactive at 60 °C [8]. Therefore, SaDAE exhibits a higher thermostability, especially at 60 and 70 °C. As shown in Fig. 1c, d. SaDAE was optimally active in Tris–HCl pH 8.0 and retained 80% of the maximal activity at pH values from 7.0 to 9.0. The enzyme was stable for 2 h at different pH values, indicating that the enzyme had a relatively extensive useful pH range for biocatalysis. The relative activity of SaDAE in the presence of various metal ions is shown in Fig. 2a. SaDAE was active even without the addition of metal ions, but was strongly enhanced by adding Mg^2+^, Co^2+^, and Mn^2+^, which enhanced its activity by 1.74-, 1.63-, and 1.34-fold, respectively. By contrast, Ni^2+^, Ca^2+^, Fe^2+^, Cu^2+^, Fe^3+^, and Zn^2+^ decreased d-allulose 3-epimerase activity to 75.6, 53.2, 48.3, 41.6, 36.0, and 25.5% of the control, respectively. The results therefore suggest that SaDAE is also a metalloenzyme, as other members of this family [4, 6]. The metal ion of Mg^2+^ greatly improved the catalytic activity toward d-fructose, which was different from family enzymes (Co^2+^ vs *C. cellulolyticum* and *Desmospora* sp. DAEases) [6, 8], and (Mn^2+^ vs *A. tumefaciens* and *C. scindens* DAEases) [4, 7].

**Fig. 3:**
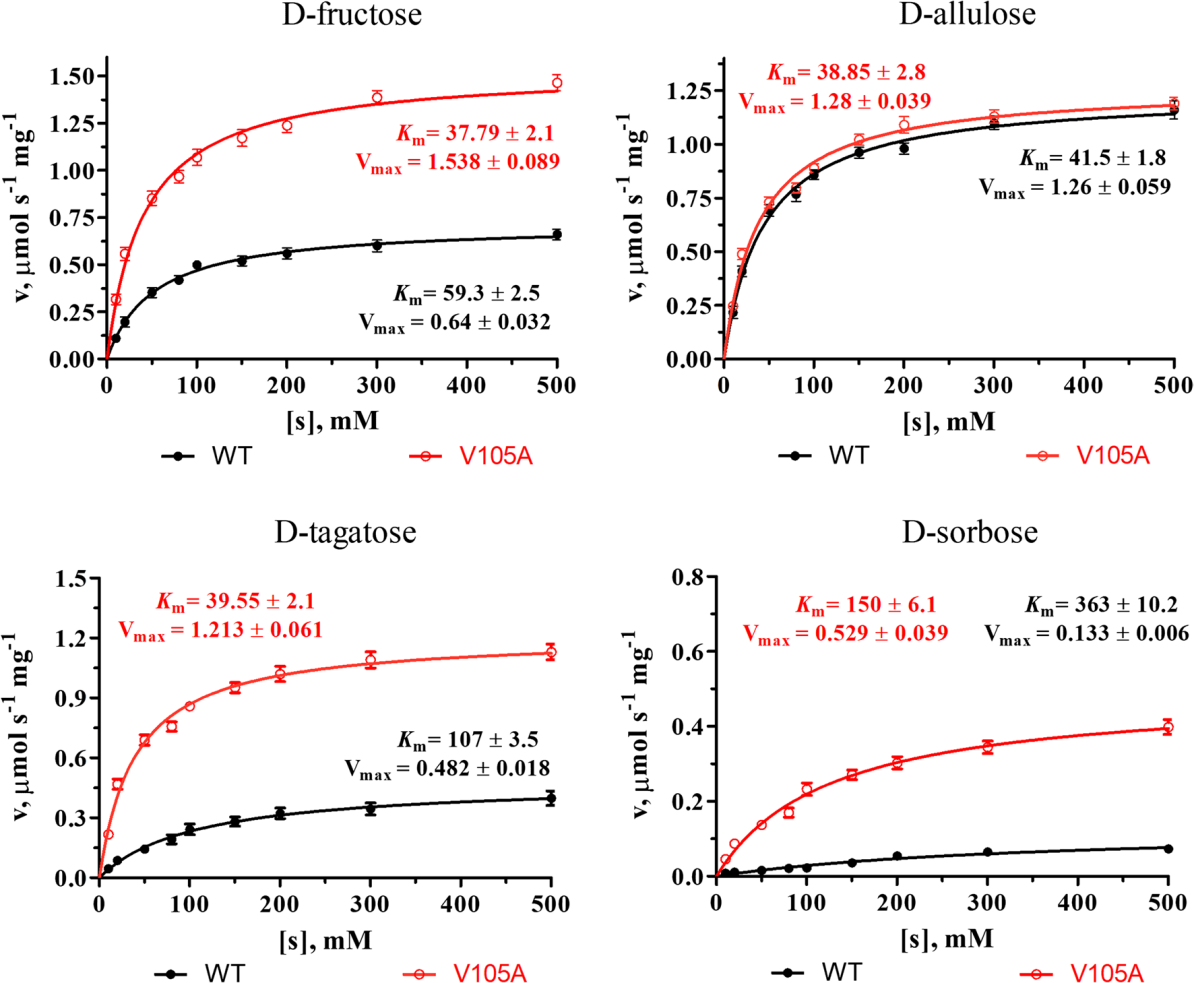
Michaelis-Menten plots of SaDAE WT and V105A toward different substrates

The SaDAE showed the highest activity toward d-allulose and moderate activity toward d-fructose and d-tagatose, respectively (Fig. 2b). The kinetic parameters of SaDAE with different substrates were determined. As shown in Fig. 3 and Table 1, the *K*_m_ values of the enzyme were between 41.5 and 363 mM, and the catalytic efficiencies (*k*_cat_*/K*_m_) were between 0.01 and 1.1 s^−1^ mM^−1^. SaDAE showed a high activity toward d-allulose with an affinity of 41.5 mM and catalytic efficiency of 1.1 s^−1^ mM^−1^. It also showed the moderate activity toward d-fructose with the affinity of 59.3 mM and catalytic efficiency of 0.39 s^−1^ mM^−1^. SaDAE showed only low catalytic activity toward d-sorbose.

**Table 1:** Kinetic parameters of SaDAE WT and V105A on four substrates

Enzymes	Substrates	*K*_m_ (mM)	*k*_cat_ (s^−1^)	*k*_cat_/*K*_m_ (s^−1^ mM^−1^)	Relative activity (%)
WT	d-Fructose	59.3 ± 2.5	22.9 ± 0.49	0.39	49.85 ± 1.36
	d-Allulose	41.5 ± 1.8	44.6 ± 0.9	1.1	100 ± 3.2
	d-Tagatose	107 ± 3.5	17.2 ± 0.65	0.16	39 ± 1.0
	d-Sorbose	363 ± 10.2	4.4 ± 0.09	0.01	8 ± 0.2
V105A	d-Fructose	37.79 ± 2.1	54.93 ± 1.6	1.45	112.78 ± 5.1
	d-Allulose	38.85 ± 2.8	45.46 ± 3.2	1.17	102.59 ± 4.6
	d-Tagatose	39.55 ± 2.1	43.32 ± 0.8	1.09	95.10 ± 3.2
	d-Sorbose	150.19 ± 6.1	18.09 ± 0.5	0.126	24.47 ± 1.3

All assays were repeated three times, and the data are shown as mean ± S.D

### Homology modeling of SaDAE

**Fig. 4:**
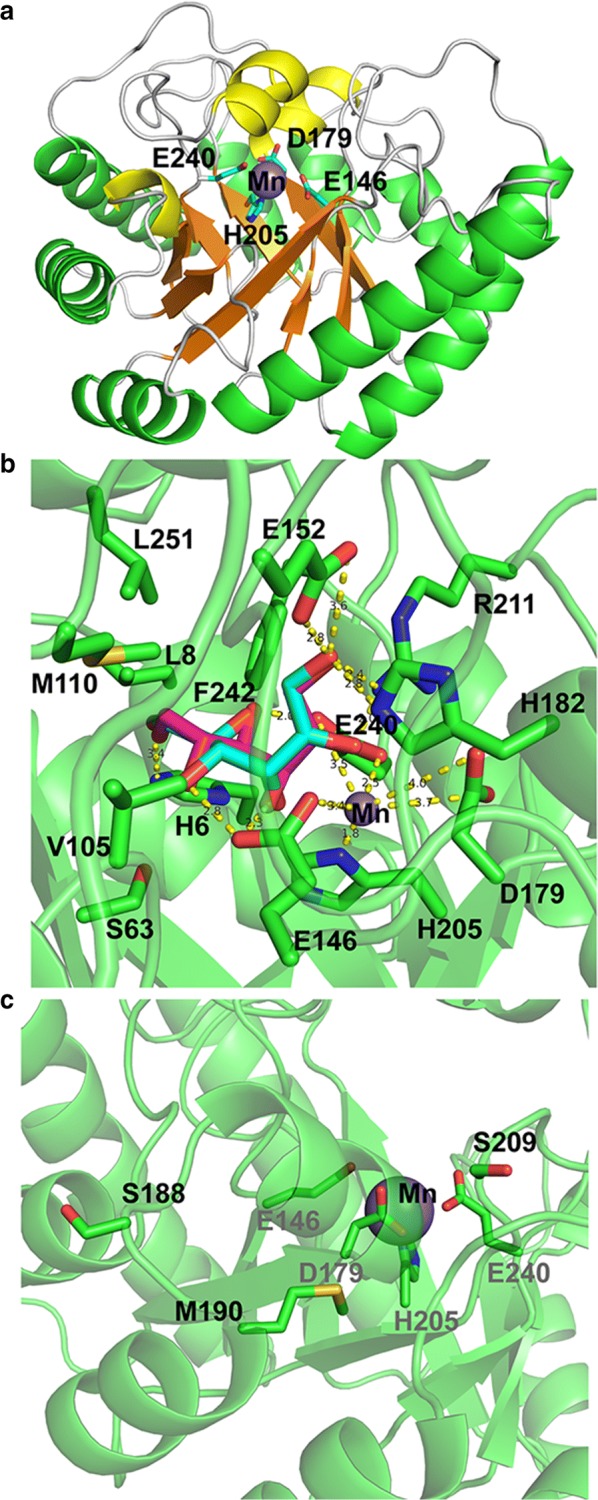
Residues at substrate binding site of SaDAE homology structure. **a** The overall structure of SaDAE. The catalytic residues are shown as cyan sticks. Mn(II) is displayed as a purple sphere. **b** Residues of SaDAE forms hydrogen bond with substrate. The substrates d-fructose and d-allulose are shown as magenta and cyan sticks, respectively. **c** The active site and rational designed residues on the surface of SaDAE

**Fig. 5:**
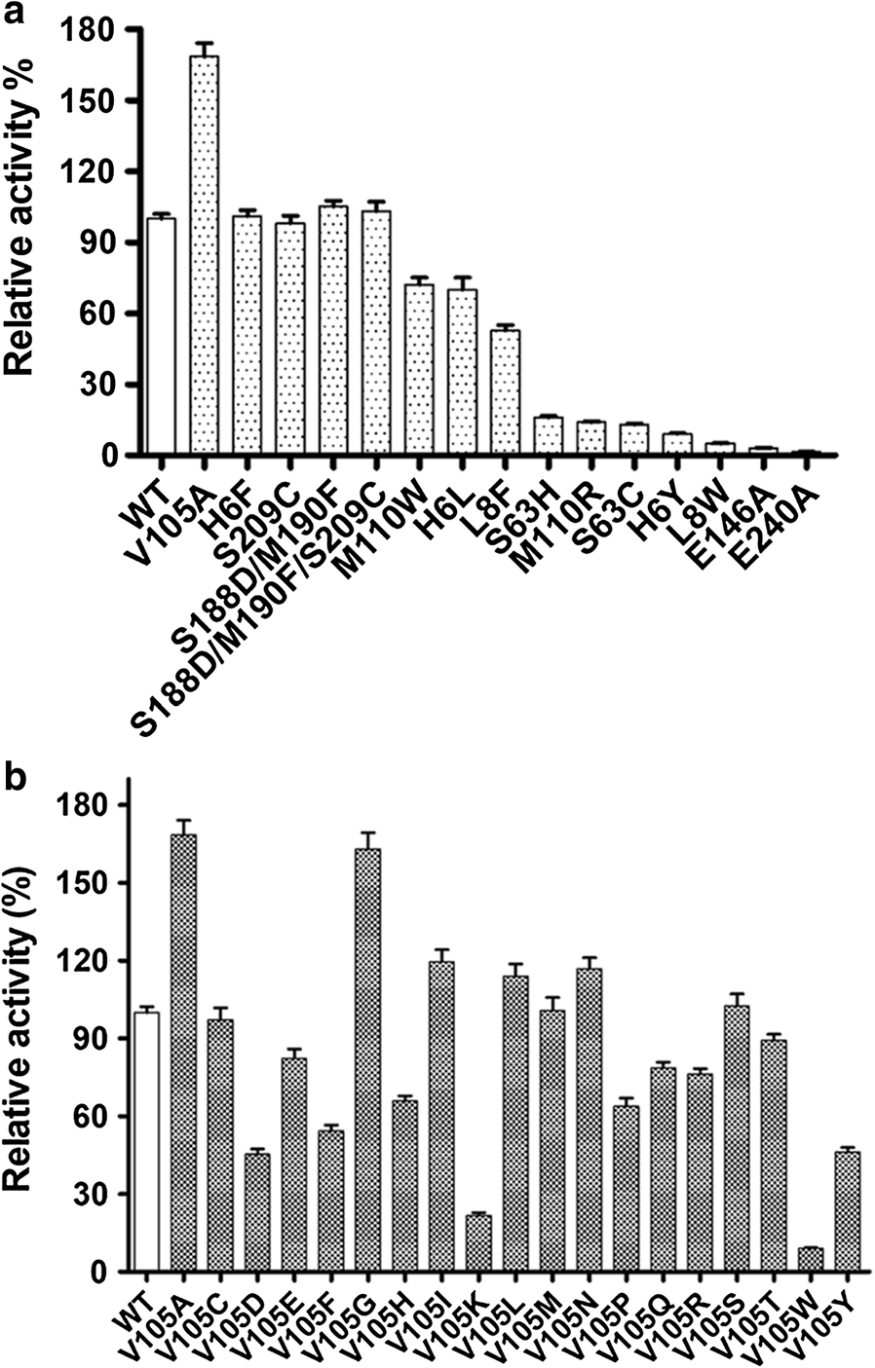
**a** The relative catalytic activity of mutant SaDAE. **b** The relative catalytic activity of saturation mutagenesis of SaDAE on V105 toward d-fructose

The homology model of SaDAE showed the characteristic TIM-barrel (β/α)_8_ fold with the center of the molecule consisting of eight repetitive units comprising a cluster of β-strand surrounded by α-helices (Fig. 4a). The α-helices of α1 and α8 were longer than *C. cellulolyticum* DAEase, *P. cichorii* DTEase, and *M. loti* LREase (Additional file 1: Figure S5), which was reported to be responsible for the thermostability of *A. tumefaciens* DAEase [14]. The loop of α2–β2 above the active site also showed the different spatial conformation. The active site comprising Glu146, Asp179, His205, and Glu240, coordinating Mn^2+^, was located at the top of TIM barrel (Fig. 4a, b). These residues form a hydrogen bond network that appears to be capable of supporting the hydride transfer reaction, suggesting that this is the catalytic tetrad of SaDAE, which is conserved in the DTE/DAE superfamily (Additional file 1: Figure S6). The three residues Glu146, Asp179, and Glu240 were completely conserved. However, His205 was not, and was exchanged for glutamate in the enzyme from *R. sphaeroides* [11]. Mn^2+^ was coordinated by the O-2 and -3 groups of the substrate d-fructose/d-allulose in a bidentate manner. These interactions form a coordination complex with a distorted octahedral geometry (Fig. 4c). This homology model supports the previously proposed reaction mechanism that proceeds via the deprotonation/protonation of the substrate at C3 by two Glu residues (Glu146 and Glu240) [13, 14, 16]. The residue Glu240, which coordinates Mn^2+^ generates a *cis*-enediolate intermediate by removing a proton from C3, after which Glu146 protonates C3 from the other side. The substitution of Glu146 and Glu240 by Ala yielded the extremely low relative activities of 3.1% and 1.5% compared with the wild-type enzyme (Fig. 5a), which indicates Glu146 and Glu240 play a vital role in the activity of SaDAE.

### Substrate specificity analysis of SaDAE

The O-1 of the substrate formed hydrogen bonds with Arg211, His182, and Glu152 (Fig. 4b). The O-2 and -3 of the substrate coordinate Mn^2+^, which neutralizes the O-2 oxyanion of a putative *cis*-enediolate. The O-3 moiety is further stabilized by hydrogen bonds with the hydroxyl groups of Glu146 and Glu240. When modeling with d-fructose or d-allulose, we found that d-fructose has the same configurations of O-1, -2, and -3 as d-allulose, although these two substrates showed the opposite steric conformation (Fig. 4b). Furthermore, the interactions between the enzyme and d-fructose/d-allulose at the O-1, -2, and -3 positions were very similar to those in other DTE/DAE family enzymes (Additional file 1: Figure S6) [13, 14, 16]. Therefore, SaDAE strictly regulates the catalytic reaction at the 1-, 2-, and 3-positions. However, the residues interacting with the O-4, -5, and -6 positions of d-fructose/d-allulose were not conserved in this family of enzymes (Additional file 1: Figure S6). The substrate-enzyme interactions at positions O-4, -5, and -6 positions are essential for substrate specificity and affinity, since they determine the spatial conformation of various substrates. In particular, Glu146 formed a unique interaction with O-4 of d-allulose and O-5 of d-fructose, while Glu240 interacted with O-5 of d-allulose and O-4 of d-fructose. Furthermore, O-6 of both substrates was stabilized by His6, which was not conserved in the DTE/DAE family. The van der Waals interaction between His6 and O-6 was found to be necessary, since the H6Y mutant lost almost all catalytic activity, while H6F retained the same level as the wild-type (Fig. 5a). Some residues, such as His9, Leu8, Val105, Met110, and Phe242 form a hydrophobic environment for substrate recognition (Fig. 4c). Notably, Met110 was positioned the same as Trp in *A. tumefaciens* DAE, *C. cellulolyticum* DAE, and *P. cichorii* DTE, while it corresponds to His in *M. loti* LRE and Arg in *R. sphaeroides* DTE. The side-chain of the Trp located in the loop of DTE/DAE family enzymes was reported to be oriented towards the substrate, and closes the active site as a lid [14]. Met110 plays a key role in substrate recognition in SaDAE, because M110W/R had decreased activities, at only 72% and 14% compared with wild-type enzyme (Fig. 5a).

Interestingly, the V105A mutant had improved activity toward d-fructose (168% of WT enzyme; Fig. 5a), and showed a high activity toward d-fructose with an affinity of 37.79 mM and catalytic efficiency of 1.45 s^−1^ mM^−1^ (Table 1 and Fig. 3), which is higher than other DAEases [4, 5, 11]. Therefore, site-directed saturation mutagenesis of Val105 was chosen as the starting point for the rational design to investigate its effect on catalytic activity based on the homology model of SaDAE. Substitution of this residue by some amino acids, such as Ala, Gly, Ile, Leu, and Asn, resulted in increased activity toward d-fructose compared to that of WT SaDAE (Fig. 5b). These mutant enzymes showed the improvement of catalytic activity of 14–68% compared with wild-type enzyme. V105S/C/M had comparable activity to that of the WT enzyme, whereas substitution with other residues exhibited 21–82% compared with that of wild-type enzyme. Furthermore, V105W showed the lowest activity, corresponding to 9% of the wild-type. The substrate specificity of the V105A was also determined (Fig. 2b and Table 1). The SaDAE V105A exhibited the higher activity toward d-fructose and d-tagatose than SaDAE WT, respectively. The biochemical data indicate that smaller side chains (Ala or Gly) at this position are very important for substrate recognition. The catalytic activities of V105A/G toward d-fructose increased by 63% and 68%, respectively (Fig. 5b).

### Biocatalytic prodution of d-allulose from d-fructose using SaDAE

**Fig. 6:**
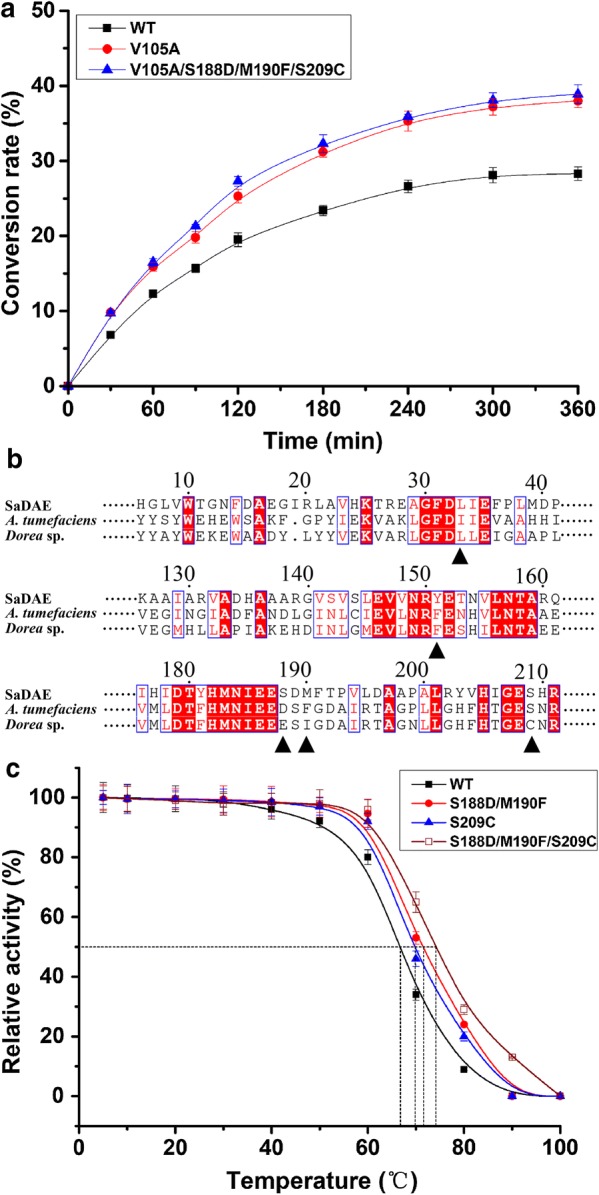
**a** Bioconversion of d-allulose by wild-type and mutant SaDAE. All assays were performed in triplicate with three independent measurements. Standard deviations of the biological replicates are represented by error bars. **b** Multiple sequence alignment of amino acid d-allulose 3-epimerase. The microorganism origins with GenBank accession numbers as follows: SaDAE: SQA09501.1; *A. tumefaciens*: AAK88700.1; *Dorea* sp.: CDD07088. **c** The *T*_m_ (50% loss of activity) of wild type and mutant SaDAE

SaDAE WT produced 142 g L^−1^ d-allulose from 500 g L^−1^ d-fructose in 6 h at pH 8.0 and 60 °C, corresponding to a conversion rate of 28.3% (Fig. 6a). Moreover, SaDAE_V105A showed a higher conversion rate of 38.9%, yielding 190 g L^−1^ d-allulose under the same conditions. This compared favorably with other enzymes from the same family, i.e. *A. tumefaciens* DAE (32.9%) [4], *P. cichorii* DTE (20%) [10], and *R. sphaeroide*s DTE (23%) [24]. In our research, both SaDAE WT and the rational designed mutants therefore showed higher biocatalytic production of d-allulose than comparable enzymes from previous studies. Rational design using site-directed mutagenesis based on structure–function relationships has improved the catalytic efficiency of SaDAE, which will aid the industrial application for rare sugars production.

### The thermostability of SaDAE and its mutants

Choi et al. reported that the I33L/S213C double-mutant of DAE from *A. tumefaciens*, which was generated using random and site-directed mutagenesis, showed a remarkably improvement of thermostability, with an increase of 7.5 °C in the melting temperature [25]. Zhang et al. redesigned the DAE from *Dorea* sp. using site-directed mutagenesis, in which the triple mutant F154Y/E191D/I193F showed greatly increased half-life (*t*_1/2_) (5.4-fold at 50 °C) and melting temperature (17.54 °C) [26]. Based on the analysis of structural homology and amino acid alignment of DAE/DTE/LRE superfamily enzymes (Figs. 4b and 6b), we redesigned SaDAE by introducing S209C, S188D/M190F, and S209C/S188D/M190F mutations to confirm their hypothesized effect on the enzyme’s thermostability. The three targeted residues are all located at the surface of the active site. With SaDAE WT, a 50% loss of activity (*T*_m_) was observed after incubation at 67 °C for 60 min, which increased to 70, 71.5, and 74.2 °C in the three mutants, respectively (Fig. 6c). Moreover, the half-life (*t*_1/2_) of the mutants at 70 °C was much longer (3.3–4.6 h) than that of the WT (2.0 h) (Table 2). Furthermore, the four-site mutation of V105A/S209C/S188D/M190F retained both the high production rate of d-allulose and high thermostability, which indicates its potential for industrial applications.

**Table 2:** The *t*_1/2_ and *T*_m_ values (50% loss of activity) of SaDAE wild-type and mutants

SaDAEase	*t*_1/2_ (h)	*T*_m_ (°C)
Wild type	2.04	67.0
S209C	3.25	70.0
S188D/M190F	3.71	71.5
S209C/S188D/M190F	4.57	74.2
V105A/S209C/S188D/M190F	4.68	74.6

## Conclusions

A novel d-allulose 3-epimerase from *Staphylococcus aureus* was found to mainly catalyse the C3-epimerization of d-fructose to d-allulose. The binding-site residues of SaDAE show a unique recognition mode at O-4, -5, and -6 of the substrate. Specifically, Glu146 was found to form a unique interaction with O-4 of d-allulose and O-5 of d-fructose, while Glu240 interacted with O-5 of d-allulose and O-4 of d-fructose. We obtained the rationally designed mutant of V105A with a higher relative activity and conversion rate towards d-fructose, and mutant S191D/M193E/S213C with higher thermostability compared with the wild-type enzyme through site-directed mutagenesis. Taken together, the data indicate that SaDAE has great potential for the production of rare sugars on the industrial scale in the future.

## Methods

### Cloning and expression of the SaDAE gene

The full-length nucleotide sequence of SaDAE (GenBank ID: UAUZ01000004.1; Protein ID: SQA09501.1) was codon-optimized for *E. coil* and ordered from Genewiz (Suzhou, China) as a synthetic DNA. The resulting synthetic DNA sequence was inserted into the vector pET28a(+) (Novagen, Madison, WI, USA) with the *Nde*I and *Eco*RI sites. The pET28a(+) plasmid with the SaDAE gene (pET28a-SaDAE) was used to transform into *E. coli* BL21(DE3), which was grown in lysogeny broth (LB) containing kanamycin (50 μg mL^−1^) at 37 °C, at a culture OD_600_ of 0.6–0.8, IPTG was added to a final concentration of 0.5 mM, followed by incubation at 16 °C overnight.

### Purification of SaDAE enzyme

The cells were harvested from the culture broth by centrifugation (5000×*g*, 15 min, 4 °C), washed twice with 0.85% saline, resuspended in lysis buffer [20 mM Tris–HCl pH 8.0, 10 mM imidazole, 500 mM NaCl, and 1 mM dithiothreitol (DTT)] containing 1 mg mL^−1^ lysozyme, and then disrupted by sonication. The cell debris was removed by centrifugation (40,000×*g*, 30 min, 4 °C). The clear supernatants were loaded onto a column containing Ni–NTA Superflow resin (Qiagen, Hilden, Germany) equilibrated with lysis buffer. The recombinant enzyme was eluted from the column using 15 mL elution buffer (20 mM Tris–HCl pH 8.0, 300 mM imidazole, 100 mM NaCl, and 1 mM DTT). The eluted solution was dialyzed against 20 mM Tris–HCl pH 8.0, 1 mM DTT overnight and was further purified by ion exchange using Source Q (column volume: 1 mL, flow rate: 3 mL min^−1^; GE Healthcare). The purified sample was transferred to a Superdex 200 Increase 10/300 GL column (GE Healthcare) in 20 mM Tris–HCl buffer pH 8.0 with 100 mM NaCl, and 1 mM DTT [27, 28]. The resulting eluate containing SaDAE was used directly in the activity assays. The total protein concentration was estimated using a BCA assay kit (Solarbio, China) and bovine serum albumin was used as the standard.

### MALDI-TOF Mass Spectrometry

A sample comprising 1 μL of the purified enzyme solution was dripped onto a MALDI target comprising 1 μL saturated alpha-cyano-4-hydroxycinnamic acid (HCCA) in a solvent mixture of acetonitrile–water (70:30, vol/vol) with 0.01% trifluoroacetic acid, and analyzed using an ultrafleXtreme TOF/TOF (Bruker Daltonics, Germany) operating in linear mode after the solutions were dried in air. FlexAnalysis software (Bruker Daltonics) was used to analyzed the MALDI-TOF spectra.

### Site-directed mutagenesis

The SaDAE mutants were constructed using plasmid SaDAE-pET28a containing the SaDAE gene as the template via a one-step PCR method, using the primers listed in Additional file 1: Table S1. The PCR program was as follows: 98 °C for 2 min, followed by 20 cycles of 98 °C for 30 s, 55 °C for 45 s, 68 °C for 6 min and 72 °C for 5 min. The PCR reaction mixtures were analyzed by agarose gel electrophoresis. *Dpn*I was used to digest the template for 60 min, and the products were used to transform *E. coli* JM109 for DNA cloning. The mutated constructs were confirmed by sequencing (Genewiz, China). The plasmids containing mutant genes were introduced into *E. coli* BL21 (DE3) for protein expression.

### CD spectroscopy

The circular dichroism (CD) spectra (MOS-450, Biologic, Claix, Charente, France) were recorded in the far-UV (190–260 nm) with a 1 mm path-length cell at 25 °C. SaDAE (0.1 mg mL^−1^) was recorded in in buffer comprising 20 mM Tris–HCl pH 8.0, 100 mM NaCl, and 1 mM DTT. Four scans were recorded using a bandwidth of 0.1 nm, a step resolution of 0.1 nm, and a scan rate of 1 nm s^−1^, and averaged for each spectrum. The protein secondary structure was analyzed using SELCON3 software (http://www.dichroweb.cryst.bbk.ac) [29].

### Confirmation of products using HPLC

The products d-allulose, d-tagatose, d-fructose, and d-sorbose were determined using high-performance liquid chromatography (HPLC) on an Agilent 1260 instrument (USA) equipped with an evaporative light-scattering detector (ELSD 6000, Alltech Technology Ltd., Canada) and a Prevail Carbohydrate ES column-W (5 μm, 4.6 × 250 mm, Agela Technologies, China), which was eluted with acetonitrile (85%) at 40 °C and 1 mL min^−1^.

### Enzyme activity assay

The enzyme activity was determined by measuring the amount of the produced d-allulose with d-fructose as the substrate. The reactions were performed for 10 min at 60 °C in a final volume of 0.5 mL Tris–HCl buffer (20 mM, pH 8.0) with 10 g L^−1^ d-fructose, 1 mM Mg^2+^, and an appropriate amount of the enzyme, unless stated otherwise. The reaction was terminated by incubation in boiling water for 5 min. The amount of the produced d-allulose was measured by HPLC. One unit of SaDAE activity was defined as the amount of enzyme that catalyzes the formation of 1 μmol d-allulose per min at pH 8.0 and 60 °C. The total protein concentration was determined using a BCA assay kit (Solarbio, China) with bovine serum albumin as the standard. All experiments were conducted in triplicate, and the data are shown as the mean ± SD.

The optimal temperature and pH for d-allulose production were determined by measuring the reaction in the range of 20 to 90 °C while the pH value was varied from 5.5 to 11 using 20 mM MES buffer (pH 5.5–6.5), 20 mM PB buffer (pH 6.0–8.0), 20 mM Tris–HCl buffer (pH 7.0–9.0), and 20 mM Glycine–NaOH buffer (pH 9.0–11.0) while the temperature was held at 70 °C for 10 min. To investigate the effect of various metal ions on the activity of SaDAE, the purified enzyme solution was treated with EDTA for 8 h and dialyzed against 20 mM Tris–HCl buffer (pH 8.0). The activity assay was conducted after the addition of 1 mM Ca^2+^, Mg^2+^, Zn^2+^, Cu^2+^, Mn^2+^, Co^2+^, Fe^2+^, and Fe^3+^, respectively. The activity without adding metal ions was defined as 100%. The pH stability of the purified enzyme was determined by incubating it at 4 °C for up to 2 h in the above-mentioned buffers. The effect of temperature on the enzyme was analyzed by incubating it in 20 mM Tris–HCl buffer (pH 8.0) at different temperatures (30, 40, 50, 60, and 70 °C) for 240 min, and sampling every 30 min. The remaining activity was measured using the same assay conditions as above. The half-times of the SaDAE wild-type and mutants were investigated using the first-order inactivation kinetic model under the optimum conditions at 70 °C. Thermal stability was determined by measuring the residual activity after the incubation at different temperatures (5–100 °C) in 20 mM Tris–HCl buffer (pH 8.0) for 60 min. The maximal SaDAE activity at 70 °C was defined as 100%. All assays were done in triplicate, and the data are shown as the mean ± SD.

### Structural modeling of SaDAE

Modeller 9.9 software was used to generate the homology model of SaDAE [30]. The crystal structure of *M. loti* l-ribulose 3-epimerase (PDB ID: 3vyl), with 44% sequence identity to SaDAE, was used as the template [31]. A sequence alignment was automatically generated between MlLRE and SaDAE using align2d command, followed by homology modeling using the automodel command. The variable target function with conjugate gradients was first used to optimize each model, followed by structure refinement using annealing via MD simulations. Finally, the values of the Modeller objective function and the DOPE assessment scores were used to choose the best model. The generated model structure was visualized and analyzed using the PyMol molecular Graphics System (http://www.pymol.org) [32].

### Biocatalytic production of d-allulose using wild-type SaDAE and its derived mutants

To examine the enzymatic conversion of d-fructose to d-allulose by the wild-type and mutant enzymes, the reaction was carried out at 60 °C in 20 mM Tris–HCl (pH 8.0), in mixtures comprising 5 µM purified SaDAE and 500 g L^−1^ d-fructose. The progress of the enzymatic conversion was measured in every hour for 6 h. The samples were diluted for HPLC analysis. Three independent measurements were performed for all reactions, which were conducted in triplicate.

## Additional file

**Additional file 1: Table S1.** Primers used for the construction of recombinant SaDAE. **Figure S1.** Multiple sequence alignment of amino acid sequence for the SaDAE with D-allulose 3-epimerase, L-ribulose 3-epimerase, and D-tagatose 3-epimerase from various strains. **Figure S2.** Purification of SaDAE by anion-exchange (a) and size-exclusion chromatography (b). Protein markers of conalbumin (75.0 kDa) and aldolase (158.0 kDa) were used. (c) MALDI-TOF spectra of SaDAE. **Figure S3.** (a) CD spectrum of SaDAE and (b) secondary structure assignments. **Figure S4.** Products confirmation of the enzymatic conversion using HPLC. (a) D-allulose (b) d-fructose (c) D-sorbose (d) D-tagatose. **Figure S5.** Structure comparison of SaDAE (yellow) with ketose 3-epimerases from different family. **Figure S6.** Multiple sequence alignment of amino acid D-allulose 3-epimerase, L-ribulose 3-epimerase, and D-tagatose 3-epimerase from different strains.

## References

[1] Emmadi M, Kulkarni SS (2014). Recent advances in synthesis of bacterial rare sugar building blocks and their applications. Nat Prod Rep.

[2] Uechi K, Takata G, Fukai Y, Yoshihara A, Morimoto K (2013). Gene cloning and characterization of L-ribulose 3-epimerase from *Mesorhizobium loti* and its application to rare sugar production. Biosci Biotechnol Biochem..

[3] Shin S-M, Cao T-P, Choi J-M, Kim S-B, Lee S-J, Lee SH, Lee D-W (2017). TM0416 is a hyperthermophilic promiscuous non-phosphorylated sugar isomerase that catalyzes various C5 and C6 epimerization reactions. Appl Environ Microbiol.

[4] Kim HJ, Hyun EK, Kim YS, Lee YJ, Oh DK (2006). Characterization of an *Agrobacterium tumefaciens*d-psicose 3-epimerase that converts d-fructose to d-psicose. Appl Environ Microbiol.

[5] Yoshihara A, Kozakai T, Shintani T, Matsutani R, Ohtani K, Iida T, Akimitsu K, Izumori K, Gullapai PK (2017). Purification and characterization of (D)-allulose 3-epimerase derived from *Arthrobacter globiformis* M30, a GRAS microorganism. J Biosci Bioeng.

[6] Mu W, Chu F, Xing Q, Yu S, Zhou L, Jiang B (2011). Cloning, expression, and characterization of a d-psicose 3-epimerase from *Clostridium cellulolyticum* H10. J Agric Food Chem.

[7] Zhang WL, Fang D, Xing QC, Zhou L, Jiang B, Mu WM (2013). Characterization of a novel metal-dependent d-psicose 3-epimerase from *Clostridium scindens* 35704. PLoS ONE.

[8] Zhang W, Fang D, Zhang T, Zhou L, Jiang B, Mu W (2013). Characterization of a metal-dependent d-psicose 3-epimerase from a novel strain, *Desmospora* sp 8437. J Agric Food Chem.

[9] Park C-S, Kim T, Hong S-H, Shin K-C, Kim K-R, Oh D-K (2016). d-Allulose production from d-fructose by permeabilized recombinant cells of *Corynebacterium glutamicum* cells expressing d-allulose 3-epimerase *Flavonifractor plautii*. PLoS ONE.

[10] Itoh H, Okaya H, Khan AR, Tajima S, Hayakawa S, Izumori K (1994). Purification and characterization of d-tagatose 3-epimerase from *Pseudomonas* sp ST-24. Biosci Biotechnol Biochem.

[11] Qi Z, Zhu Z, Wang J-W, Li S, Guo Q, Xu P, Lu F, Qin H-M (2017). Biochemical analysis and the preliminary crystallographic characterization of d-tagatose 3-epimerase from *Rhodobacter sphaeroides*. Microb Cell Fact.

[12] Zhu Z, Li C, Liu X, Gao D, Wang X, Tanokura M, Qin H-M, Lu F (2019). Biochemical characterization and biocatalytic application of a novel d-tagatose 3-epimerase from *Sinorhizobium* sp. RSC Adv..

[13] Chan H-C, Zhu Y, Hu Y, Ko T-P, Huang C-H, Ren F, Chen C-C, Ma Y, Guo R-T, Sun Y (2012). Crystal structures of d-psicose 3-epimerase from *Clostridium cellulolyticum* H10 and its complex with ketohexose sugars. Protein Cell..

[14] Kim K, Kim H-J, Oh D-K, Cha S-S, Rhee S (2006). Crystal structure of d-psicose 3-epimerase from *Agrobacterium tumefaciens* and its complex with true substrate d-fructose: a pivotal role of metal in catalysis, an active site for the non-phosphorylated substrate, and its conformational changes. J Mol Biol.

[15] Yoshida H, Yoshihara A, Gullapalli PK, Ohtani K, Akimitsu K, Izumori K, Kamitori S (2018). X-ray structure of *Arthrobacter globiformis* M30 ketose 3-epimerase for the production of d-allulose from d-fructose. Acta Crystallogr F..

[16] Yoshida H, Yamada M, Nishitani T, Takada G, Izumori K, Kamitori S (2007). Crystal structures of d-tagatose 3-epimerase from *Pseudomonas cichorii* and its complexes with d-tagatose and d-fructose. J Mol Biol.

[17] Kuhlman B, Dantas G, Ireton GC, Varani G, Stoddard BL, Baker D (2003). Design of a novel globular protein fold with atomic-level accuracy. Science.

[18] Kiss G, Çelebi-Ölçüm N, Moretti R, Baker D, Houk K (2013). Computational enzyme design. Angew Chem Int Ed.

[19] Qin H-M, Zhu Z, Ma Z, Xu P, Guo Q, Li S, Wang J-W, Mao S, Liu F, Lu F (2017). Rational design of cholesterol oxidase for efficient bioresolution of cholestane skeleton substrates. Sci Rep..

[20] Qin H-M, Miyakawa T, Nakamura A, Hibi M, Ogawa J, Tanokura M (2014). Structural optimization of SadA, an Fe(II)- and α-ketoglutarate-dependent dioxygenase targeting biocatalytic synthesis of *N*-succinyl-l-threo-3,4-dimethoxyphenylserine. Biochem Biophys Res Commun.

[21] Bosshart A, Panke S, Bechtold M (2013). Systematic optimization of interface interactions increases the thermostability of a multimeric enzyme. Angew Chem Int Ed.

[22] Zhang W, Jia M, Yu S, Zhang T, Zhou L, Jiang B, Mu W (2016). Improving the thermostability and catalytic efficiency of the d-psicose 3-epimerase from *Clostridium bolteae* ATCC BAA-613 using site-directed mutagenesis. J Agric Food Chem.

[23] Mu WM, Zhang WL, Fang D, Zhou L, Jiang B, Zhang T (2013). Characterization of a d-psicose-producing enzyme, d-psicose 3-epimerase, from *Clostridium* sp. Biotechnol Lett.

[24] Zhang L, Mu W, Jiang B, Zhang T (2009). Characterization of d-tagatose-3-epimerase from *Rhodobacter sphaeroides* that converts d-fructose into d-psicose. Biotechnol Lett.

[25] Choi J-G, Ju Y-H, Yeom S-J, Oh D-K (2011). Improvement in the thermostability of d-psicose 3-epimerase from *Agrobacterium tumefaciens* by random and site-directed mutagenesis. Appl Environ Microbiol.

[26] Zhang W, Zhang Y, Huang J, Chen Z, Zhang T, Guang C, Mu W (2018). Thermostability improvement of the d-allulose 3-epimerase from *Dorea* sp. CAG317 by site-directed mutagenesis at the interface regions. J Agric Food Chem..

[27] Qin HM, Miyakawa T, Inoue A, Nishiyama R, Nakamura A, Asano A, Sawano Y, Ojima T, Tanokura M (2017). Structure and polymannuronate specificity of a eukaryotic member of polysaccharide lyase family 14. J Biol Chem.

[28] Qin H-M, Miyakawa T, Inoue A, Nishiyama R, Nakamura A, Asano A, Ojima T, Tanokura M (2018). Structural basis for controlling the enzymatic properties of polymannuronate preferred alginate lyase FlAlyA from the PL-7 family. Chem Commun.

[29] Whitmore L, Wallace BA (2008). Protein secondary structure analyses from circular dichroism spectroscopy: methods and reference databases. Biopolymers.

[30] Šali A, Blundell TL (1993). Comparative protein modelling by satisfaction of spatial restraints. J Mol Biol.

[31] Uechi K, Sakuraba H, Yoshihara A, Morimoto K, Takata G (2013). Structural insight into l-ribulose 3-epimerase from *Mesorhizobium loti*. Acta Crystallogr D..

[32] Humphrey W, Dalke A, Schulten KVMD (1996). Visual molecular dynamics. J Mol Graph.

