# Supplementary material for: Redesign of a novel d-allulose 3-epimerase from Staphylococcus aureus for thermostability and efficient biocatalytic production of d-allulose
Source: Microb Cell Fact. 2019 Mar 25;18:59. doi: 10.1186/s12934-019-1107-z (PMC6432756; doi:10.1186/s12934-019-1107-z)
Supplement: Supplementary file 1 — Additional file 1: Table S1. Primers used for the construction of recombinant SaDAE. Figure S1. Multiple sequence alignment of amino acid sequence for the SaDAE with D-allulose 3-epimerase, L-ribulose 3-epimerase, and D-tagatose 3-epimerase from various strains. Figure S2. Purification of SaDAE by anion-exchange (a) and size-exclusion chromatography (b). Protein markers of conalbumin (75.0 kDa) and aldolase (158.0 kDa) were used. (c) MALDI-TOF spectra of SaDAE. Figure S3. (a) CD spectrum of SaDAE and (b) secondary structure assignments. Figure S4. Products confirmation of the enzymatic conversion using HPLC. (a) D-allulose (b) d-fructose (c) D-sorbose (d) D-tagatose. Figure S5. Structure comparison of SaDAE (yellow) with ketose 3-epimerases from different family. Figure S6. Multiple sequence alignment of amino acid D-allulose 3-epimerase, L-ribulose 3-epimerase, and D-tagatose 3-epimerase from different strains. [file 12934_2019_1107_MOESM1_ESM.docx]

Additional Information for

Redesign of a novel D-allulose 3-epimerase from *Staphylococcus aureus* for thermostability and efficient biocatalytic production of D-allulose

Zhangliang Zhu^a^, Dengke Gao^a^, Chao Li^a^, Ying Chen^a^, Menglu Zhu^a^, Xin Liu^a^, Masaru Tanokura^a,b*^, Hui-Min Qin^a*^, Fuping Lu^a*^

^a^Key Laboratory of Industrial Fermentation Microbiology of the Ministry of Education; Tianjin Key Laboratory of Industrial Microbiology; College of Biotechnology, Tianjin University of Science and Technology; National Engineering Laboratory for Industrial Enzymes; Tianjin 300457, P. R. China

^b^ Laboratory of Basic Science on Healthy Longevity, Department of Applied Biological Chemistry, Graduate School of Agricultural and Life Sciences, The University of Tokyo, 1-1-1 Yayoi, Bunkyo, Tokyo 113-8657, Japan

*Corresponding authors: College of Biotechnology, Tianjin University of Science and Technology, M. Tanokura: amtanok@mail.ecc.u-tokyo.ac.jp; H.-M. Qin: huiminqin@tust.edu.cn; F. Lu: lfp@tust.edu.cn;

Tel: +86-22-60601958. Fax: +86-22-60602298

**Table S1. Primers used for the construction of recombinant SaDAE.**

| Primer name | Sequence of primer |
| --- | --- |
| H6F_F | CGGCAGCCATATGAATATCGGCTGT**GCT**GGTCTGGTGTGG |
| H6F_R | CCACACCAGACC**AGC**ACAGCCGATATTCATATGGCTGCCG |
| H6L_F | TATGAATATCGGCTGT**CTT**GGTCTGGTGTGGACGG |
| H6L_R | CGGTCCACACCAGACC**AAG**ACAGCCGATATTCATA |
| H6Y_F | CATATGAATATCGGCTGT**TAT**GGTCTGGTGTGGACCG |
| H6Y_R | CGGTCCACACCAGACC**ATA**ACAGCCGATATTCATATG |
| L8W_F | aatatcggctgtcacggt**tgg**gtgtggacgggtaac |
| L8W_R | gttacccgtccacac**cca**accgtgacagccgatatt |
| L8F_F | atcggctgtcacggt**ttc**gtgtggacgggtaac |
| L8F_R | gttacccgtccacac**gaa**accgtgacagccgat |
| S63C_F | ggcggttagcgcg**tgt**ctgggtctgag |
| S63C_R | ctcagacccaga**cac**gcgctaaccgcc |
| S63H_F | tggcggttagcgcg**cat**ctgggtctgagcg |
| S63H_R | cgctcagacccaga**tgc**gcgctaaccgcca |
| V105A_F | gagcatttttgcggc**gcg**atctacagcgcgatg |
| V105A_R | catcgcgctgtagat**cgc**gccgcaaaaatgctc |
| V105C_F | ggcgagcatttttgcggc**tgc**atctacagcgcgatgaag |
| V105C_R | cttcatcgcgctgtagat**gca**gccgcaaaaatgctcgcc |
| V105D_F | gcgagcatttttgcggc**gat**atctacagcgcgatgaag |
| V105D_R | cttcatcgcgctgtagat**atc**gccgcaaaaatgctcgc |
| V105E_F | gagcatttttgcggc**gag**atctacagcgcgatg |
| V105E_R | catcgcgctgtagat**ctc**gccgcaaaaatgctc |
| V105F_F | gcgagcatttttgcggc**ttc**atctacagcgcgatgaa |
| V105F_R | ttcatcgcgctgtagat**gaa**gccgcaaaaatgctcgc |
| V105G_F | gagcatttttgcggc**ggg**atctacagcgcgatg |
| V105G_R | catcgcgctgtagat**ccc**gccgcaaaaatgctc |
| V105H_F | ggcgagcatttttgcggc**cat**atctacagcgcgatgaag |
| V105H_R | cttcatcgcgctgtagat**atg**gccgcaaaaatgctcgcc |
| V105I_F | ggcgagcatttttgcggc**ata**atctacagcgcgatgaag |
| V105I_R | cttcatcgcgctgtagat**tat**gccgcaaaaatgctcgcc |
| V105K_F | gcgagcatttttgcggc**aag**atctacagcgcgatga |
| V105K_R | tcatcgcgctgtagat**ctt**gccgcaaaaatgctcgc |
| V105L_F | cgagcatttttgcggc**ttg**atctacagcgcgat |
| V105L_R | atcgcgctgtagat**caa**gccgcaaaaatgctcg |
| V105M_F | cgagcatttttgcggc**atg**atctacagcgcgat |
| V105M_R | atcgcgctgtagat**cat**gccgcaaaaatgctcg |
| V105N_F | gtggcgagcatttttgcggc**aat**atctacagcgcgatgaagaa |
| V105N_R | ttcttcatcgcgctgtagat**att**gccgcaaaaatgctcgccac |
| V105P_F | cgagcatttttgcggc**ccg**atctacagcgcgatg |
| V105P_R | catcgcgctgtagat**cgg**gccgcaaaaatgctcg |
| V105Q_F | cgagcatttttgcggc**cag**atctacagcgcgatg |
| V105Q_R | catcgcgctgtagat**ctg**gccgcaaaaatgctcg |
| V105R_F | gcgagcatttttgcggc**agg**atctacagcgcgatga |
| V105R_R | tcatcgcgctgtagat**cct**gccgcaaaaatgctcgc |
| V105S_F | gcgagcatttttgcggc**tcg**atctacagcgcgatga |
| V105S_R | tcatcgcgctgtagat**cga**gccgcaaaaatgctcgc |
| V105T_F | gcgagcatttttgcggc**acg**atctacagcgcgatga |
| V105T_R | tcatcgcgctgtagat**cgt**gccgcaaaaatgctcgc |
| V105W_F | gcgagcatttttgcggc**tgg**atctacagcgcgatga |
| V105W_R | tcatcgcgctgtagat**cca**gccgcaaaaatgctcgc |
| V105Y_F | gtggcgagcatttttgcggc**tat**atctacagcgcgatgaagaa |
| V105Y_R | ttcttcatcgcgctgtagat**ata**gccgcaaaaatgctcgccac |
| M110W_F | ggcgtgatctacagcgc**gtg**gaagaaatacatggagcc |
| M110W_R | ggctccatgtatttcttc**cac**gcgctgtagatcacgcc |
| M110R_F | cgtgatctacagcgc**gag**gaagaaatacatggagc |
| M110R_R | gctccatgtatttcttc**ctc**gcgctgtagatcacg |
| E146A_F | tgttagtgttagcctc**gca**gtggtgaaccgctatg |
| E146A_R | catagcggttcacca**ctg**cgaggctaacactaaca |
| S188D_F | cacatgaacatcgaggag**gac**gacatgtttacgccggt |
| S188D_R | accggcgtaaacatgtc**gtc**ctcctcgatgttcatgtg |
| M190F_F | catcgaggagagcgac**ttc**tttacgccggttctgg |
| M190F_R | ccagaaccggcgtaaa**gaa**gtcgctctcctcgatg |
| S209C_F | gcacatcggcgag**tgt**catcgcggcta |
| S209C_R | tagccgcgatg**aca**ctcgccgatgtgc |
| E240A_F | gtccaatcgtgttc**gcg**agctttagcagcgc |
| E204A_R | gcgctgctaaagct**cgc**gaacacgattggac |


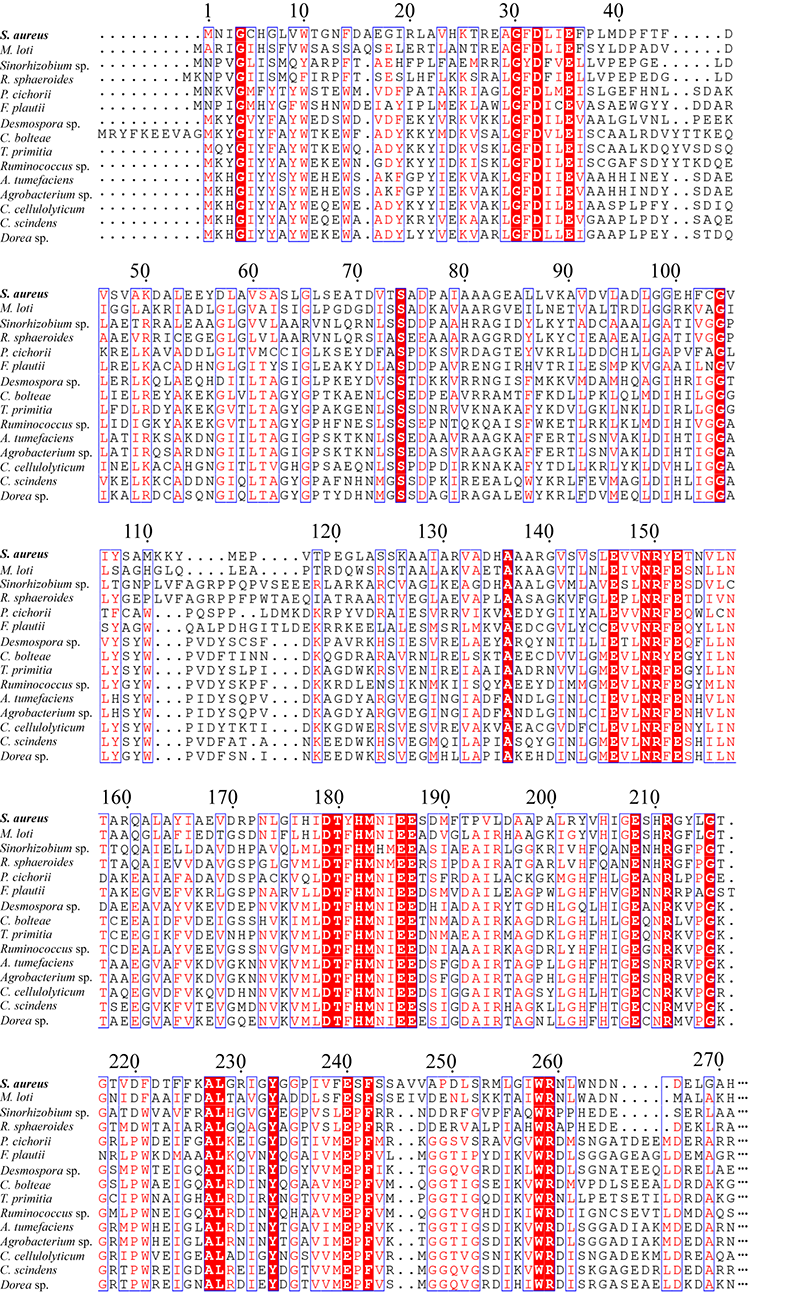


**Figure S1** Multiple sequence alignment of amino acid sequence for the SaDAE with D-allulose 3-epimerase, L-ribulose 3-epimerase, and D-tagatose 3-epimerase from various strains. Sequence alignment was performed using Clustal X and ESPript 3.0. Conserved sequences are indicated with boxes, and similar sequences are indicated using a colored background. The microorganism origins with GenBank accession numbers as follows: *S. aureus*: SQA09501.1; *M. loti*: BAB50456.1; *Sinorhizobium* sp.: WP_069063284.1; *R. sphaeroides*: ACO59490.1; *P. cichorii*: BAA24429.1; *F. plautii*: EHM40452.1; *Desmospora* sp.: WP_009711885.1; *C. bolteae*: EDP19602.1; *T. primitia*: ZP_09717154.1; *Ruminococcus* sp.: ZP_04858451.1; *A. tumefaciens*: AAK88700.1; *Agrobaxterium* sp.: EGL65884.1; *C. cellulolyticum*: ACL75304.1; *C. scindens*: EDS06411.1; *Dorea* sp.: CDD07088.


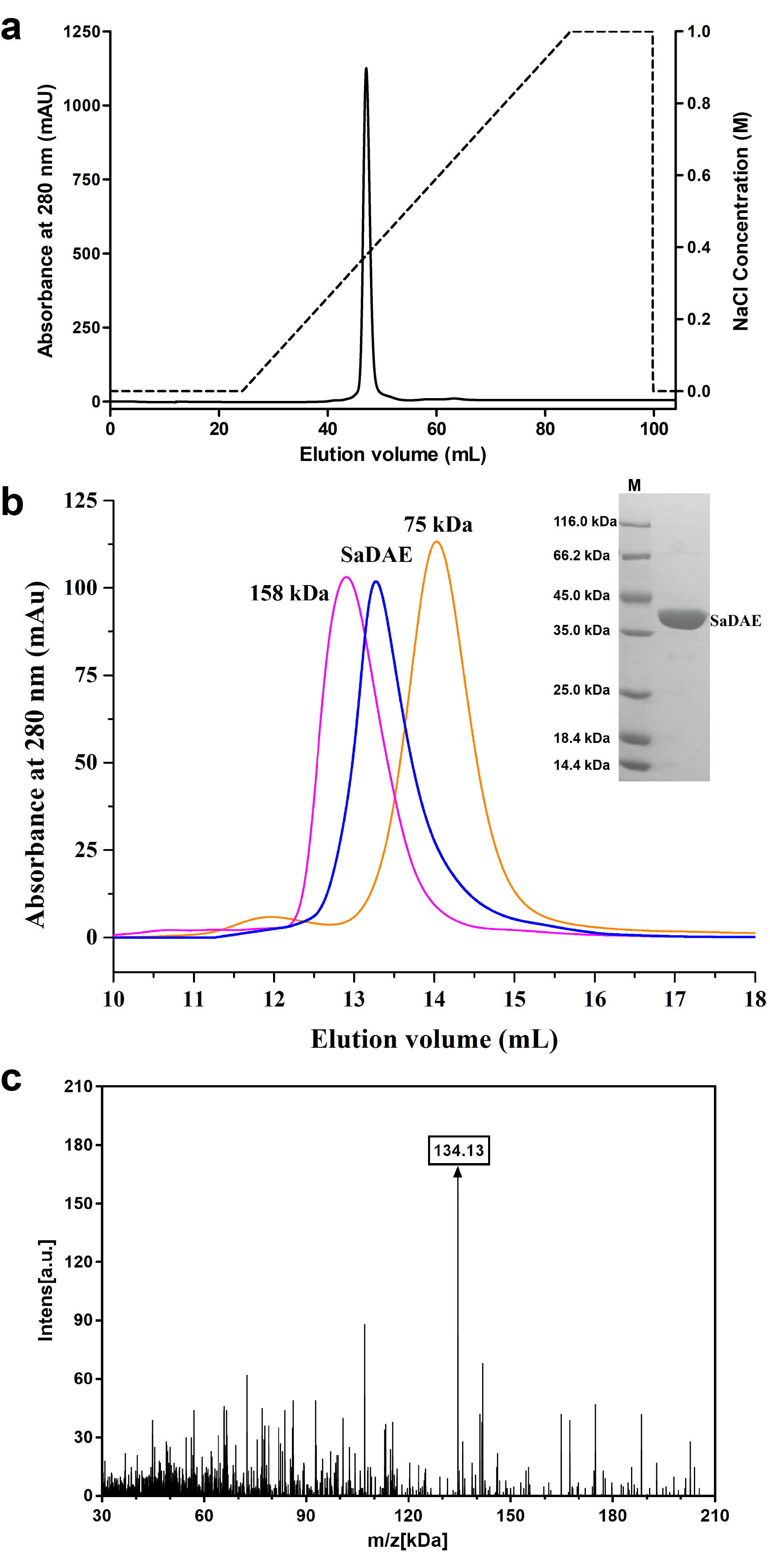


**Figure S2.** Purification of SaDAE by anion-exchange (a) and size-exclusion chromatography (b). Protein markers of conalbumin (75.0 kDa) and aldolase (158.0 kDa) were used. (c) MALDI-TOF spectra of SaDAE.


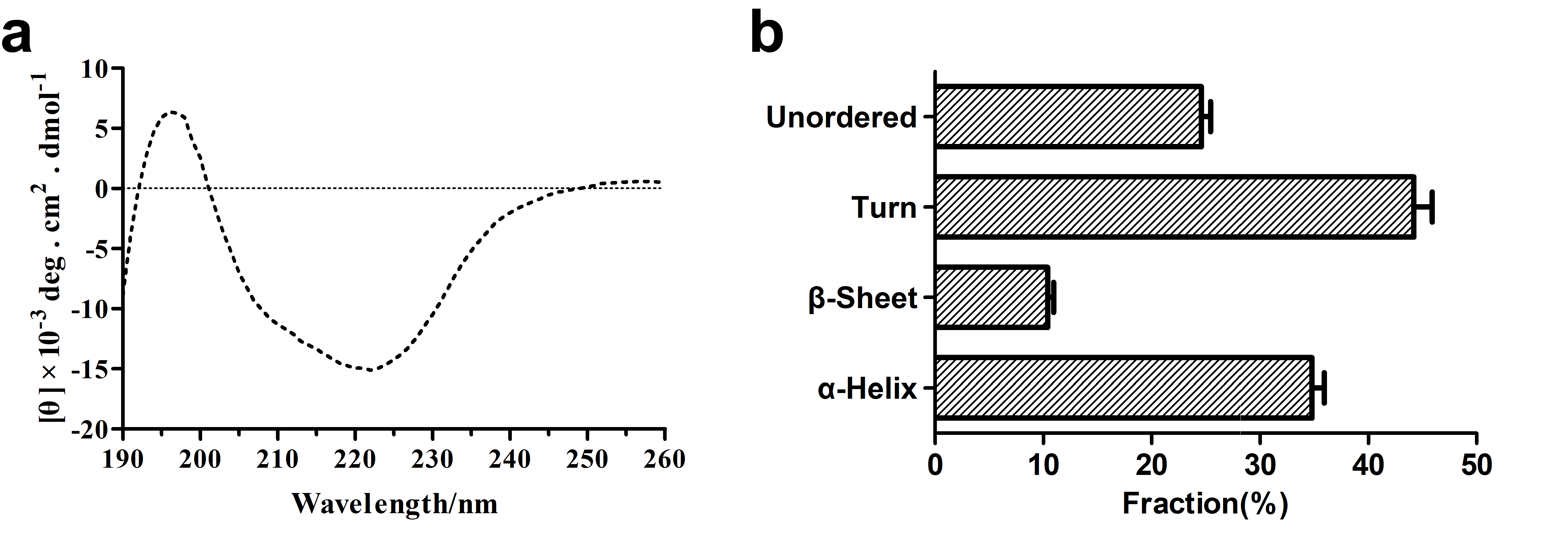


**Figure S3** (a) CD spectrum of SaDAE and (b) secondary structure assignments.

**
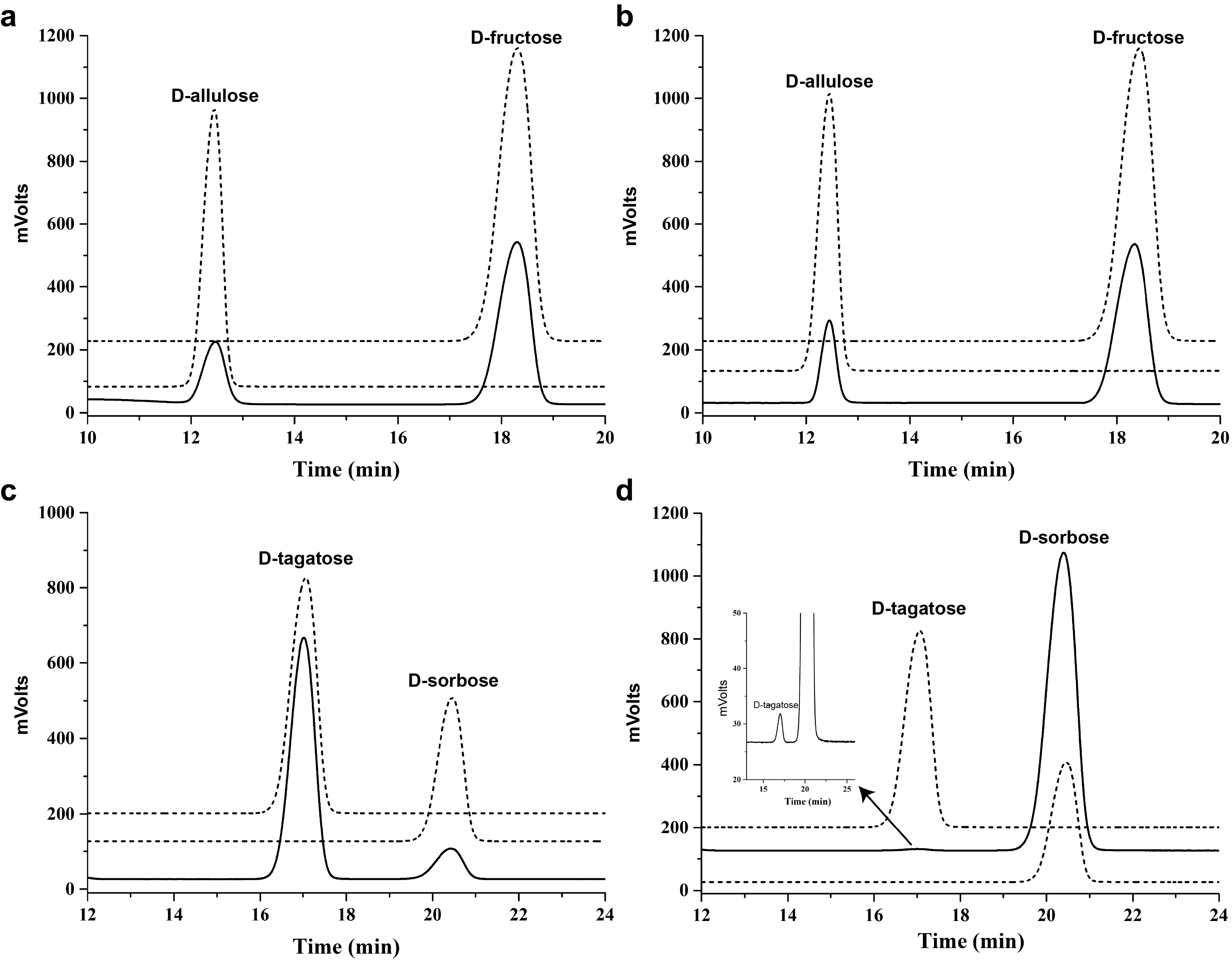
**

**Figure S4.** Products confirmation of the enzymatic conversion using HPLC. (a) D-allulose (b) D-fructose (c) D-sorbose (d) D-tagatose.

**
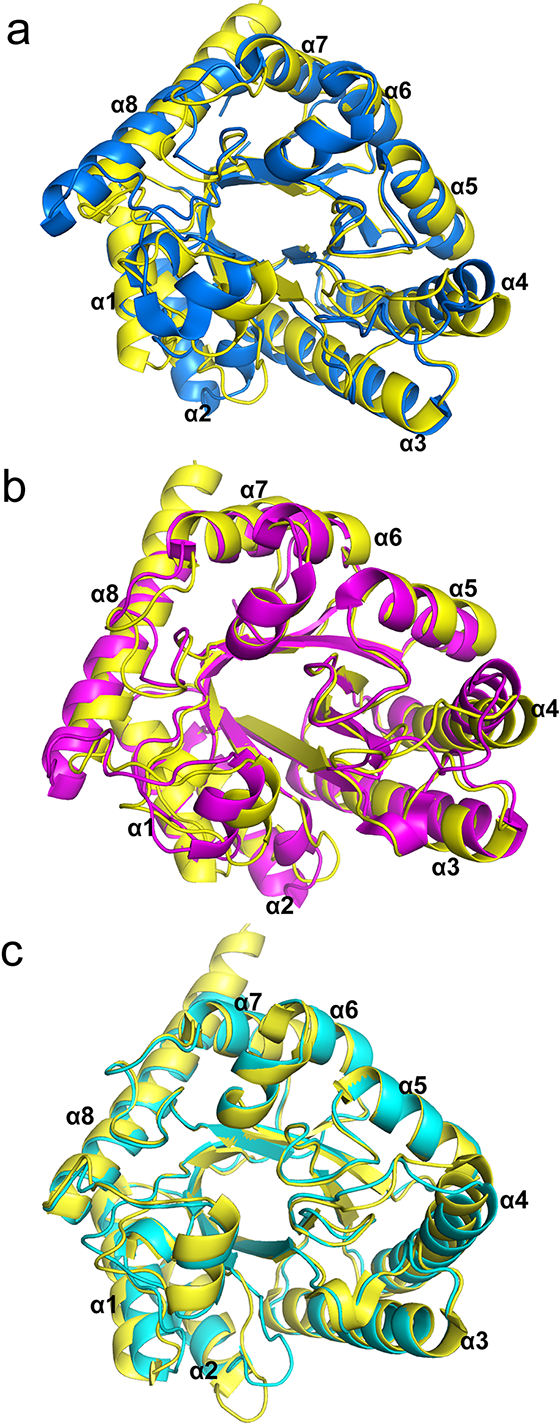
**

**Figure S5** Structure comparison of SaDAE (yellow) with ketose 3-epimerases from different family. (a) D-allulose 3-epimerase family: *C. cellulolyticum* DAEase (PDB: 3vni; blue) (b) D-tagatose 3-epimerase family: *P. cichorii* DTEase (PDB: 2qul; magenta) (c) L-ribulose 3-epimerase family: *M. loti* LREase (PDB: 3vyl; cyan), α-helices of ketose 3-epimerases were numbered from α1 to α8.

**
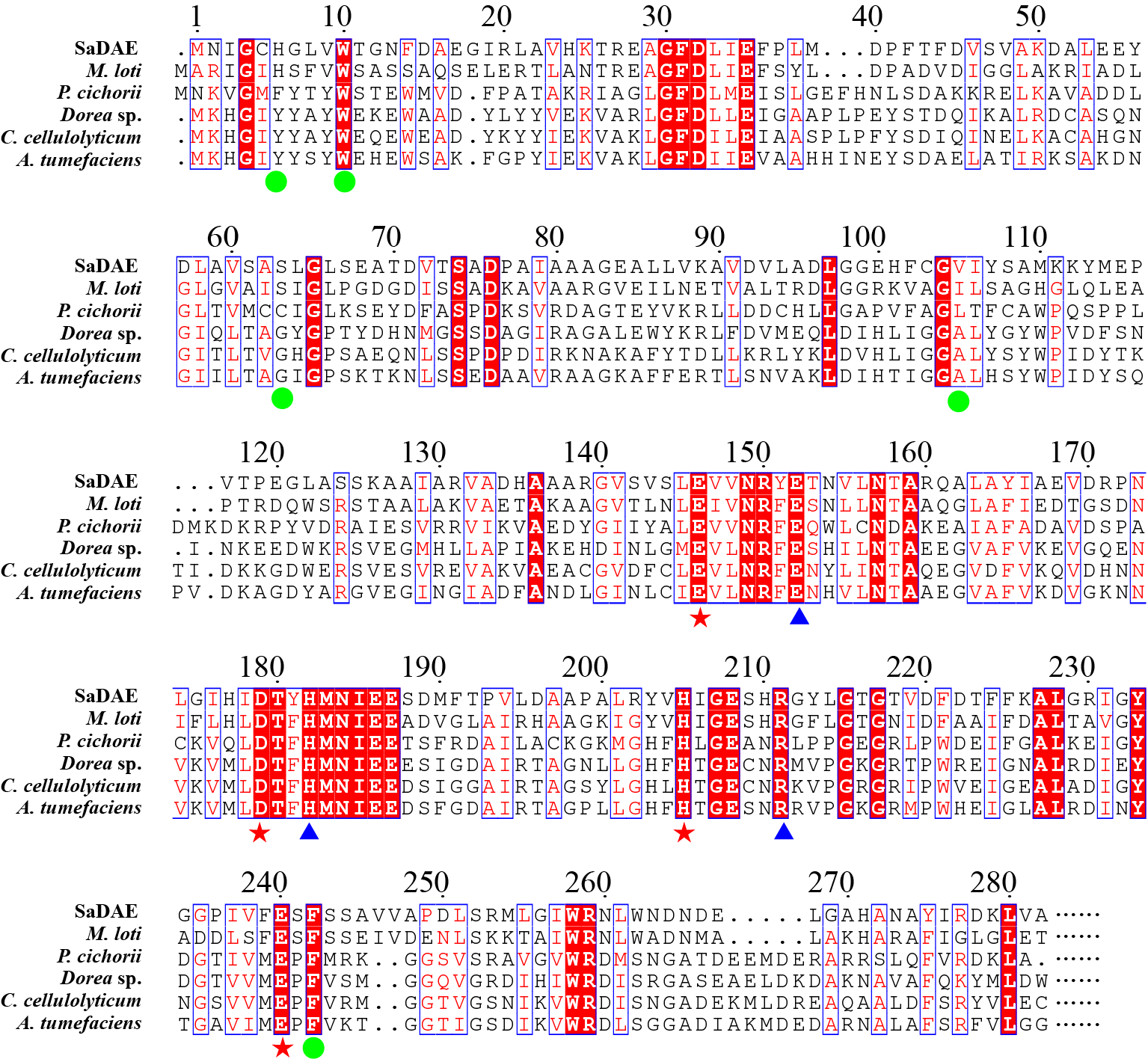
**

**Figure S6** Multiple sequence alignment of amino acid D-allulose 3-epimerase, L-ribulose 3-epimerase, and D-tagatose 3-epimerase from different strains. The residues involved in the metal coordinating site are marked as red asterisk, residues responsible for the interaction between the enzyme and O-1, -2, and -3 of D-fructose are marked as blue triangle and those between the enzyme and O-4, -5, and -6 of D-fructose are marked as green circle. The microorganism origins with GenBank accession numbers as follows: SaDAE: SQA09501.1; *M. loti*: BAB50456; *P. cichorii*: BAA24429.1; *Dore* sp.: CDD07088; *C. cellulolyticum*: ACL75304.1; *A. tumefaciens*: AAK88700.1.
